# Perfusion and diffusion MRI combined with ^11^C-methionine PET in the preoperative evaluation of suspected adult low-grade gliomas

**DOI:** 10.1007/s11060-013-1178-3

**Published:** 2013-06-16

**Authors:** Shala Ghaderi Berntsson, Anna Falk, Irina Savitcheva, Andrea Godau, Maria Zetterling, Göran Hesselager, Irina Alafuzoff, Elna-Marie Larsson, Anja Smits

**Affiliations:** 1 Department of Neuroscience, Neurology, Uppsala University, University Hospital, 751 85 Uppsala, Sweden; 2 Department of Radiology, Neuroradiology, Uppsala University, Uppsala, Sweden; 3 Department of Nuclear Medicine, PET Centre, University Hospital, Uppsala, Sweden; 4 Department of Neuroscience, Neurosurgery, Uppsala University, Uppsala, Sweden; 5 Department of Pathology, Neuropathology, Uppsala University, Uppsala, Sweden

**Keywords:** Low-grade gliomas, Perfusion-MRI, Diffusion-MRI, Positron emission tomography (PET), ^11^C-methionine (MET), Preoperative evaluation

## Abstract

Perfusion and diffusion magnetic resonance imaging (pMRI, dMRI) are valuable diagnostic tools for assessing brain tumors in the clinical setting. The aim of this study was to determine the correlation of pMRI and dMRI with ^11^C-methionine positron emission tomography (MET PET) in suspected low-grade gliomas (LGG) prior to surgery. Twenty-four adults with suspected LGG were enrolled in an observational study and examined by MET PET, pMRI and dMRI. Histological tumor diagnosis was confirmed in 23/24 patients (18 gliomas grade II, 5 gliomas grade III). The maximum relative cerebral blood volume (rCBV_max_) and the minimum mean diffusivity (MD_min_) were measured in tumor areas with highest MET uptake (hotspot) on PET by using automated co-registration of MRI and PET scans. A clearly defined hotspot on PET was present in all 23 tumors. Regions with rCBV_max_ corresponded with hotspot regions in all tumors, regions with MD_min_ corresponded with hotspot regions in 20/23 tumors. The correlation between rCBV_max_ (r = 0.19, *P* = 0.38) and MD_min_ (r = −0.41, *P* = 0.053) with MET uptake in the hotspot was not statistically significant. Taken into account the difficulties of measuring perfusion abnormalities in non-enhancing gliomas, this study demonstrates that co-registered MET PET and pMRI facilitates the identification of regions with rCBV_max_. Furthermore, the lack of a clear positive correlation between tumor metabolism in terms of MET uptake and tumor vascularity measured as rCBV_max_ suggests that combined pMRI/PET provides complementary baseline imaging data in these tumors.

## Introduction

Low-grade gliomas (LGG) in adults refer to a heterogeneous group of primary brain tumors classified as astrocytomas, oligodendrogliomas, and oligoastrocytomas World Health Organization (WHO) grade II [1]. LGG grow slowly and patients have a highly variable prognosis depending on clinical prognostic factors as well as specific histological and molecular tumor characteristics [2]. However, most if not all LGG progress to high-grade gliomas (HGG) with eventually fatal outcome.

The optimal timing of treatment for LGG remains controversial [3]. A particular challenge is the interpretation of stable disease. From a radiological point of view, there is no stable disease. LGG expand continuously during the clinically stable phase of disease, with a radiological mean growth rate of ~4 mm/year before malignant transformation [4]. Already during the initial “silent” period when these tumors may be discovered incidentally, there is a continuous growth at a rate similar to that during later phases [5]. The symptomatic phase is usually initiated by new-onset seizures, occurring in 70–90 % of all patients [6]. Focal neurologic deficits as initial symptoms are less common, probably related to the slow tumor growth, allowing functional compensation through brain plasticity mechanisms [7].

Taking into account the slow but continuous growth of LGG, early surgical resection is recommended for patients with operable tumors [7]. For patients with inoperable tumors or tumors in eloquent areas, a “watchful waiting” strategy with regular clinical and imaging surveillance may be adopted. These patients will be subjected to diagnostic biopsy and adjuvant radio- and/or chemotherapy when signs of more aggressive tumor growth occur. Thus, there is a need for sensitive imaging techniques to identify patients at risk for early tumor progression amongst suspected LGG.

Morphological MRI shows anatomical tumor location, tumor size, and contrast enhancement at presentation and is used to determine the individual tumor growth rate by repeated volumetric measurements over time [8]. Physiological MRI such as perfusion (pMRI) and diffusion (dMRI) imaging provide additional information on tumor vascularity and cellularity [9–11]. To date, perfusion and diffusion parameters are no reliable preoperative predictors of tumor grade in suspected LGG due to overlapping values between tumors with different tumor grades and histological subtypes [12]. However, these methods have become valuable diagnostic tools for LGG in the clinical setting.

Positron emission tomography (PET) with the tracer ^11^C-methionine (MET) is another valuable method in the clinical management of LGG that allows evaluation of metabolic tumor activity [13, 14]. The uptake of the amino acid tracer MET is up-regulated in glioma capillaries [15] and correlates with increased cell proliferation and tumor malignancy grade [16]. MET accumulates markedly in malignant gliomas but accumulates also in the absence of contrast enhancement [17, 18]. MET PET is used to differentiate between neoplastic and non-neoplastic lesions, for stereotactic biopsies guidance [19], and as a prognostic marker for LGG [20]. For these purposes, MET PET is performed as part of the preoperative diagnostic work-up at our hospital in all suspected LGG.

In this observational study, we investigated a series of 24 adults presenting with suspected LGG by pMRI and dMRI, in addition to MET PET. The study design was set to reflect the clinical situation at our center where decisions by the neuro-oncology team are based on clinical parameters, morphological MRI and MET PET. Our aim was to study the correlation of pMRI and dMRI with MET PET in this population prior to histopathological diagnosis.

## Materials and methods

### Patient population

Twenty-four patients (>18 year) presenting with suspected LGG referred to the Neurosurgery Department, Uppsala University Hospital, between February 2010 and September 2012 were enrolled and investigated by morphological MRI, pMRI and dMRI according to the study protocol. The institutional review board approved the study and written informed consent was obtained prior to participation. Inclusion criteria were clinical and morphological MRI findings suggestive of LGG, and a maximum time interval between PET and MRI of 90 days. Radiological diagnosis was based on typical appearance on morphological MRI with T1-weighted MRI showing no or minimal contrast enhancement. Clinical exclusion criteria were the presence of major neurological or cognitive deficits suggestive of HGG. MET PET findings were incorporated in clinical decisions to obtain early histology-proven diagnosis but high MET uptake suggestive of HGG did not exclude patients from the study [14].

### MRI techniques

Morphological MRI sequences with a 3T scanner (Philips Achieva, Best, the Netherlands) included sagittal and axial T2-weighted turbo spin echo (SE), coronal and axial T2-weighted fluid attenuated inversion recovery (FLAIR), axial T1-weighted SE before and after contrast injection, sagittal T1-weighted 3D turbo field echo after contrast injection.

pMRI was acquired with a gradient echo echo-planar imaging (EPI) sequence and dynamic susceptibility contrast-enhanced (DSC) technique as previously described [21]. Relative cerebral blood volume (rCBV) maps were calculated based on established tracer kinetic models applied to first pass data using commercial perfusion analysis software (Nordic Ice, Nordic NeuroLab, Bergen, Norway) [9, 22, 23].

dMRI was acquired using a SE EPI sequence with the following scan parameters: TR/TE = 6,700/77 ms; slice thickness/gap, 2 mm/2 mm, diffusion gradient encoding in 48 directions, b = 1,000 s/mm^2^. Mean diffusivity (MD) maps were obtained after automatic pixel-by-pixel calculation in the scanner as previously described [24].

PET and MRI images of each case were co-registered using an automated procedure in a Picture Archiving and Communication System Carestream (PACS) (Carestream, Rochester, NY, USA) to ensure precise anatomical comparability of MET PET and MRI [25, 26].

### MRI analysis

We performed two different sets of perfusion measurements. First, estimations of the average intratumoral perfusion were made on rCBV maps only, independently of MET PET scans. A large circular or oval region of interest (ROI) was placed in each tumor on the transverse rCBV map, co-registered with the T2 FLAIR slice with the largest tumor diameter (mean ROI area 307 mm^2^), avoiding large blood vessels, cysts, necrosis, and susceptibility artifacts. The rCBV ratio was calculated by dividing the mean rCBV in this ROI with the mean rCBV within a ROI of similar size in the corresponding location of the contralateral hemisphere.

Secondly, perfusion and diffusion measurements were obtained from co-registered MRI/PET scans. The maximum relative cerebral blood volume (rCBV_max_) and minimum mean diffusivity (MD_min_) were measured by first placing a 20 mm^2^ circular ROI on the rCBV map and on the MD map in the region corresponding to the PET hotspot area. The ROI was then systematically moved outside the hotspot region in all tumor slices in order to measure regions with visually suspected high perfusion and low diffusion, respectively, and to obtain rCBV_max_ and MD_min_ values for each case. The rCBV_max_ ratio was calculated by dividing the mean rCBV_max_ in the tumor ROI by the mean rCBV in a similar ROI located in normal appearing white matter of the contralateral hemisphere. MD was also measured in contralateral white matter in 20 mm^2^ ROIs to ensure that measured values were within those of normal brain tissue.

### PET technique

Patients were evaluated prior to treatment that could interfere with metabolism, except for antiepileptic drugs, and fasted 4 h before tracer injection. Twelve patients were examined by ECAT EXACT HR + camera (Siemens/CTI, Knoxville) with an axial field of view (FOV) of 15.5 cm, providing 63 contiguous 2.43 mm slices. In 12 patients, a Discovery ST (GE Healthcare, Milwaukee) PET/CT scanner was used with a 15.7 cm axial FOV providing 47 contiguous 3.27 mm slices. PET scanning was performed as described previously [26]. The two PET cameras were carefully calibrated to ensure interchangeable test results and stable tracer kinetics in the acquisition time period [27].

### PET analysis

Analysis of PET data was performed using Voyager 4 software (GE Healthcare, Uppsala). ROIs were manually defined with knowledge of the results of the morphological MRI. A tumor ROI was drawn around the lesion on the brain slice containing the five hottest pixels of the lesion (hotspot). The mean peak pixel value over the five connected pixels, representing an area of about 20 mm^2^, defined the maximum MET uptake in the hotspot. To obtain a tumor-to-normal background ratio, the MET uptake in the tumor was related to the uptake in a cortical reference region located in the contralateral hemisphere at the axial plane of the thalamus [28]. This hotspot/contralateral cortex ratio (HS/cortex ratio) was used for all statistical analyses. In case of heterogeneously enhanced MET uptake in the tumor, multiple hotspots were delineated separately and the highest HS/cortex ratio was used.

### Histopathology

Histological diagnosis was obtained by resection (*n* = 17) or biopsy (*n* = 7). All biopsies were directed to MET hotspot regions, except for one patient with a hotspot in the primary motor cortex. In this case, biopsy was derived from the region adjacent to the hotspot region. The neuropathologist re-evaluated all clinical diagnoses for the purpose of the study according to WHO criteria [1]. The presence of loss of heterozygosity on chromosomal arms 1p and 19q (LOH 1p/19q) in tumors with an oligodendroglial component was examined by microsatellite technique as previously described [29].

### Statistical analysis

Analysis of the correlation between rCBV_max_, MD_min_ and maximum MET uptake in histologically proven tumors (*n* = 23) was performed by Spearman correlation test. Differences in HS/cortex ratios, rCBV_max_ and MD_min_ values between tumors of different grade and histological subtype were assessed by non-parametric test (Mann–Whitney). A *P* value <0.05 was considered statistically significant. Statistical analysis was performed using IBM SPSS Inc (version 19).

## Results

### Patients

Twenty-four patients (12 males, 12 females) with mean age of 48.9 years (SD ± 15.2, range 22–78 years) at radiological diagnosis were included. Twenty-two patients (92 %) presented with epileptic seizures and received antiepileptic drugs, two patients with minor neurological symptoms such as dizziness and headache. Clinical, histological and radiological tumor characteristics are summarized in Table 1.

**Table 1:** Clinical characteristics, PET and MRI findings of the entire study sample (*n* = 24)

Patient/ sex/age	Histology	Tumor location	Tumor size diameter (mm) L × W × H	Contrast-enhancement (CE)	MET uptake ratio in HS	MD_min_* value in PET HS	rCBV_max_ ratio in PET HS	rCBV ratio in large ROI
1/M/62	OAII	Parietal/R	37 × 32 × 28	No CE	2.6	1.22	2.51	1.19
2/M/67	AII	Frontal/L	38 × 27 × 32	No CE	1.6	1.25	1.47	0.28
3/M/59	GGII	Temporal/L	25 × 27 × 14	No CE	3.1	1.07	1.93	1.27
4F/40	OII	Frontal/R	41 × 23 × 38	No CE	1.7	1.25	2.61	1.82
5/F/53	Encephalitis	Temporal/L	12 × 13 × 12	No CE	1.2	0.84	0.92	0.84
6/M/32	OAIII	Temporal/L	76 × 37 × 42	Minimal	2.9	1.03	3.44	0.98
7/F/36	AIII	Parietal/L	28 × 44 × 55	No CE	2.9	1.37	8.27	0.64
8/F/31	OII	Frontal/R	46 × 32 × 37	Minimal	3.1	0.85	6.59	4.24
9/F/32	OII	Frontal/R	34 × 38 × 35	No CE	1.8	1.01	2.36	0.88
10/M/25	OII	Parietal/L	42 × 24 × 25	No CE	1.6	1.25	3.73	0.99
11/F/78	AIII	Frontal/L	29 × 28 × 21	No CE	2.7	0.96	4.14	0.79
12/M/54	AII	Frontal/L	56 × 25 × 27	No CE	3.5	0.92	3.53	1.33
13/F/63	AII	Temporal/R	45 × 32 × 36	Minimal	3.8	0.98	3.51	1.19
14/F/22	OII	Fronto-parietal/R	58 × 29 × 37	No CE	1.7	0.99	4.64	0.38
15/M/70	AIII	Temporal/R	71 × 30 × 31	Minimal	2.1	0.65	3.35	0.98
16/F/40	OIII	Parietal/R	44 × 38 × 46	No CE	2.0	1.11	3.81	0.62
17/M/52	OAII	Parietal/R	33 × 21 × 23	No CE	3.1	0.75	1.18	0.63
18/M/41	AII	Temporal/L	41 × 25 × 29	No CE	2.2	1.06	1.55	0.46
19/F/43	AII	Fronto-temporal/L	45 × 40 × 34	No CE	1.3	1.90	2.4	0.55
20/M/67	AII	Temporo-Parietal	66 × 51 × 48	No CE	2.1	1.28	3.07	0.95
21/F/43	OII	Frontal/R	59 × 49 × 58	Minimal	2.0	0.69	2.57	0.62
22/F/65	AII	Temporal/L	51 × 49 × 40	No CE	1.3	1.12	1.55	0.85
23/M/33	OAII	Frontal/L	32 × 34 × 33	Minimal	2.8	1.19	3.8	0.66
24/M/60	AII	Frontal/L	40 × 26 × 34	No CE	1.3	1.03	3.76	0.71

*AII/III* astrocytoma grade II/III, *OAII* oligoastrocytoma grade II, *OII/III* oligodendroglioma grade II/III, *GGII* ganglioglioma grade II. Tumor location: *R* right, *L* left; L × W × H; length × width × height; ×10^−3^mm^2^/s

### Histological diagnosis

Tumor diagnosis was confirmed in 23 patients (Table 1). Tumor diagnoses consisted of glioma grade II (*n* = 18; 8 astrocytomas grade II; 6 oligodendrogliomas grade II; 3 oligoastrocytomas grade II; 1 ganglioglioma grade II) and glioma grade III (*n* = 5; 3 astrocytomas grade III; 1 oligodendroglioma grade III; 1 oligoastrocytomas grade III) (Table 1). Microsatellite analysis confirmed the presence of LOH 1p/19q in 9/11 tumors (82 %) with an oligodendroglial component. Patient 5 had biopsies from six different locations in the tumor-suspected area without histological proof of tumor and was diagnosed with non-specified encephalitis.

### Time interval between PET, MRI, and surgery

The mean time interval between PET and MRI was 9 days (0–85 days). MET PET was performed on the same day as MRI (*n* = 7) or within 1 month from MRI (*n* = 15). For two patients, this time interval was 46 respectively 85 days. The mean time interval between completed MRI/PET imaging and surgery was 70 days (1 day–323 days). Eleven patients underwent surgery within 4 weeks, eight patients within 3 months, and five patients within 5–11 months after completed imaging. All five patients with delayed surgery (5–11 months) showed clinically stable disease during the entire observation period.

### MET PET

All except one patient (23/24) had a clearly defined hotspot on PET. The majority of cases showed one single hotspot (*n* = 17), others two separate hotspots (*n* = 6) or three separate hotspots (*n* = 1). In one tumor without a clear hotspot (patient 22), the ROI was moved within the tumor to obtain maximum MET uptake ratios for statistical analysis. HS/cortex ratios varied from 1.2–3.8 for the whole sample. The mean HS/cortex ratio for gliomas grade II (*n* = 18) was 2.25 ± 0.80 (SD) and for gliomas grade III (*n* = 5) 2.52 ± 0.43 (SD).

### Morphological MRI

The radiological tumor characteristics of the entire sample are summarized in Table 1. All lesions had supratentorial location and involved cortical brain areas. Six tumors showed minimal contrast enhancement on T1-weighted images.

### Perfusion MRI

**Fig. 1:**
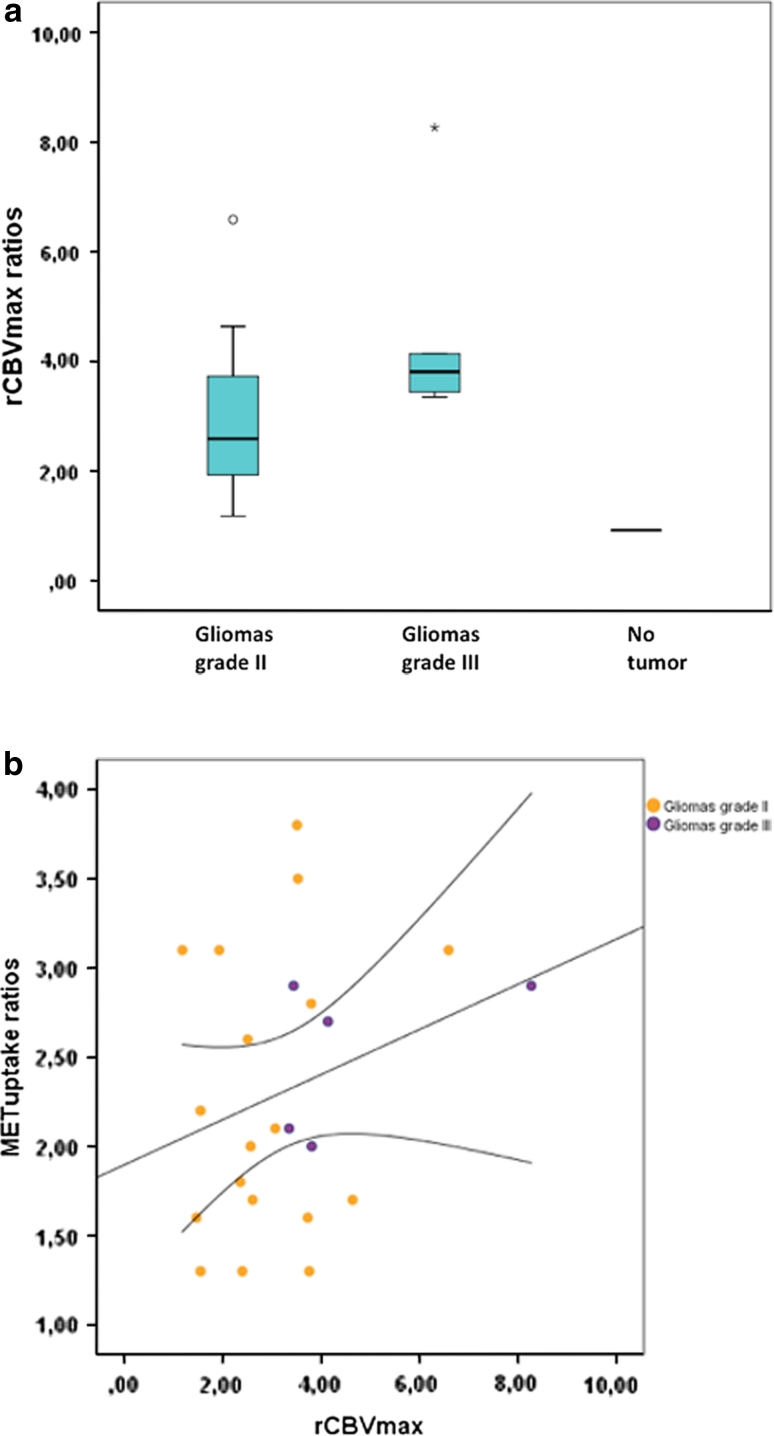
**a** *Box plots* showing the distribution of rCBV_max_ ratios for the entire sample (*n* = 24). **b** *Scatter plot* showing the correlation between maximum MET uptake (HS/cortex ratio) and rCBV_max_ values in PET hotspot regions in all tumor samples (*n* = 23). *Straight line*, *regression line*, *dotted lined*: 95 % CI

The rCBV_max_ ratios in the PET hotspot and the rCBV ratios in the large tumor ROI for each case (*n* = 24) are shown in Table 1. The mean rCBV_max_ in PET hotspot regions for the whole sample was 3.19 ± 1.66 (range 0.92–8.27) (Fig. 1a). The mean rCBV in the large tumor ROI was 0.99 ± 0.77 (range 0.28–4.24), which was significantly lower (*P* = 0.0001). Regions with rCBV_max_ corresponded to hotspot regions on PET in all cases. The correlation between rCBV_max_ ratios in the hotspot region and HS/cortex ratios is shown in Fig. 1b (Spearman’s r = 0.19, *P* = 0.38). There was a significant difference in mean rCBV_max_ between gliomas grade II and gliomas grade III (*P* = 0.04), but not between enhancing and non-enhancing lesions (*P* = 0.34).

### Diffusion MRI

**Fig. 2:**
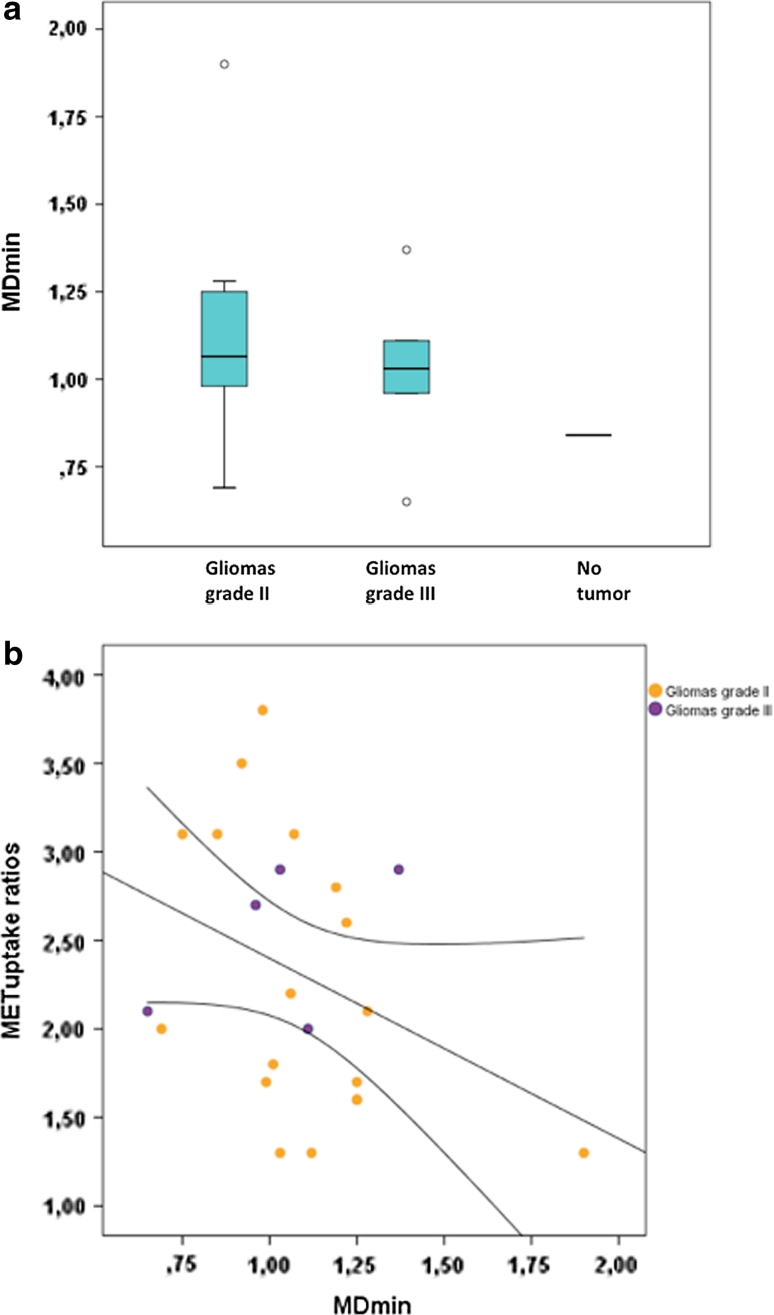
**a** *Box plots* showing the distribution of MD_min_ values for the entire sample (*n* = 24). **b** *Scatter plot* showing the correlation between maximum MET uptake (HS/cortex ratio) and MD_min_ values (×10^−3^ mm^2^/s) in PET hotspot regions in all tumor samples (*n* = 23). *Straight line*, *regression line*, *dotted lined*: 95 % CI

MD_min_ values in tumors were equal or higher than in contralateral normal appearing white matter (data not shown), in agreement with previous studies [11, 30]. MD_min_ values in MET hotspot regions varied between 0.65 and 1.9 × 10^−3^mm^2^/s (mean 1.0 ± 0.25) (Fig. 2a). In four lesions, MD_min_ values were measured in non-hotspot tumor regions (mean 1.0 ± 0.21 × 10^−3^ mm^2^/s; range 0.71–1.17). MD_min_ values between gliomas grade II and gliomas grade III were not significantly different (*P* = 0.10) (Fig. 2a). The mean MD_min_ for oligodendroglial tumors harboring LOH 1p/19q (*n* = 9) was lower than for those with intact 1p/19q (*n* = 2), but the difference was not significant (*P* = 0.81). Figure 2b shows the correlation between MD_min_ values in the hotspot region and HS/cortex ratios (Spearman’s r = −0.41, *P* = 0.053).

## Discussion

**Fig. 3:**
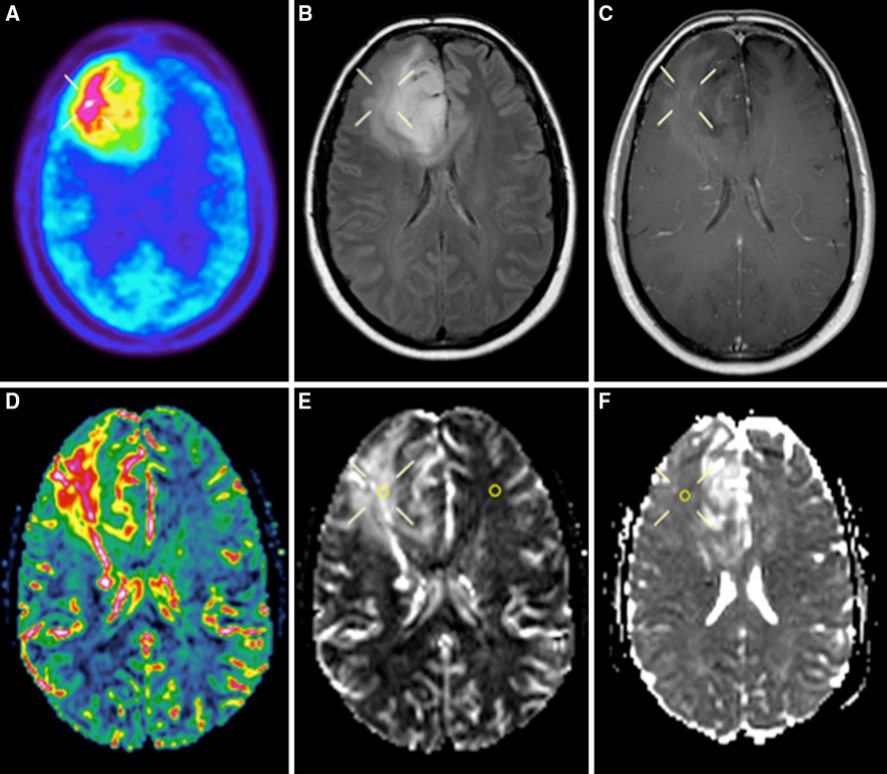
Patient 8. Preoperative MET PET and MRI of a right-sided frontal oligodendroglioma grade II in a 31-year-old female. **a** MET PET shows the hotspot region of the tumor. **b** T2-weighted FLAIR MRI shows a high signal intensity tumor. **c** T1-weighted contrast-enhanced MRI shows minimal contrast enhancement in the tumor area. **d** DSC perfusion MRI with rCBV color map shows high perfusion in the tumor area. **e** DSC perfusion MRI with rCBV *grey-scale* map shows high perfusion in the region corresponding to the PET hotspot. The ROIs in the tumor and in the contralateral normal appearing white matter are marked. **f** dMRI where the MD_min_ value in the region corresponding to the PET hotspot is lower than MD in the medial portion of the tumor (but minimally increased compared to normal appearing contralateral white matter)

In the present study, we investigated 24 patients with suspected LGG by morphological MRI, physiological MRI and MET PET prior to surgery. Our aim was to define the correlation between MET uptake and perfusion and diffusion abnormalities in these tumors at the time point of radiological diagnosis. We found that regions with rCBV_max_ corresponded with hotspot regions on PET in all tumors. However, in spite of a consistent topographical overlap between hotspot on MET PET and areas with maximum tumor perfusion, there was no significant positive correlation between rCBV_max_ and highest MET uptake. Our results are in contrast to a previous study, reporting a positive correlation between rCBVmax and maximum MET uptake and indicating a close link between amino acid transport in the tumor and vascularity [31]. This discrepancy may be partly due to the different study populations and methodologies, but also to the general difficulties of measuring subtle differences in regional perfusion in non-enhancing infiltrating tumors [32]. It is difficult to select a ROI with high intra- and inter-observer reproducibility in LGG, since perfusion images are noisy compared to both morphological images and MET PET scans. Susceptibility artifacts with signal loss and adjacent signal increase, simulating perfusion increase, may also disturb perfusion images. In addition, high perfusion values in vessels and adjacent cortex may be difficult to differentiate from increased tumor perfusion. Therefore, pMRI alone appears to be unsuited to replace MET PET in providing reliable baseline data on tumor vascularity. This is further illustrated by our perfusion measurements calculated within a large tumor area and without prior knowledge of MET PET, which were significantly lower than the co-registered PET/MRI measurements. The present study shows that the co-registered measurement of rCBV_max_ and MET uptake is a reliable way to define perfusion abnormalities in LGG (illustrated in Fig. 3).

The inclusion criteria for presumed LGG in this study are in accordance with the traditional imaging view of these tumors as non- or slightly enhancing tumors. The absence of contrast enhancement is not specific for LGG, and the degree of the blood–brain barrier disruption is not considered diagnostic for either high- or low-grade tumors [33]. In agreement with previous reports, three non-enhancing lesions in our study were anaplastic gliomas while four tumors with minimal contrast enhancement were LGG [34]. Contrast enhancement is not equivalent with perfusion abnormality, which reflects the degree of microvascularization and as such is a better indicator of the biologic aggressiveness of the tumor [21]. In a retrospective study of both LGG and HGG, baseline rCBV was a prognostic predictor for progression-free survival [35]. This is an important observation, indicating that pMRI, similar to MET PET, can be used to guide timing of treatment in suspected LGG. Our study suggests that combined pMRI/PET provides complementary baseline imaging data on tumor metabolism and vascularity that could be subsequently used in the follow-up of LGG. Longitudinal studies of patients with LGG are needed to determine the specific clinical applications of these methods as prognostic and/or predictive biomarkers.

HGG usually have higher rCBV_max_ than LGG, but rCBV_max_ may also be elevated in LGG. Oligodendroglial tumors with their characteristic dense networks of capillary branches have a generally higher rCBV_max_ than astrocytic tumors [12]. The evaluation of rCBV in oligodendrogliomas is further complicated by the fact that tumors with co-deletions of chromosome 1p/19q show generally higher rCBV_max_ than non-deleted oligodendrogliomas [36]. In our relatively small study sample, we found no significant difference between mean rCBV_max_ of astrocytic and oligodendroglial tumors (data not shown) [37].

We found a negative correlation between MET uptake in the hotspot and MD_min_ values, although the correlation did not reach significance. High MET uptake and restricted diffusion are both suggestive of more malignant tumor portions, but a relationship between MET uptake and MD_min_ has not been previously reported [10, 11]. Tumor diffusion as measured by dMRI correlates with tumor cell density and is decreased in malignant gliomas due to a higher cellularity with restricted motion of water molecules in the extracellular space. In spite of generally lower MD_min_ values in HGG, dMRI is not useful for preoperative grading due to the marked overlap in MD values between gliomas of different histological subtype and grade [30, 38, 39]. Interestingly, a recent report showed that information on anisotropy provided by diffusion tensor imaging is more useful than MD_min_ values for predicting tumor malignancy [39].

The present study has some limitations apart from the small sample size. In two patients, the time interval between PET and MRI investigation exceeded 1 month. Keeping in mind the continuous growth of LGG, it is important to perform both imaging studies simultaneously if possible.

## Conclusion

Hotspot regions on MET PET corresponded with maximum tumor perfusion and mainly low diffusion in non- or minimal enhancing gliomas. MET PET facilitated the detection of representative tumor regions with perfusion abnormalities prior to surgery, and combined pMRI/PET may provide useful as a baseline investigation in the long-term follow-up of LGG.
